# A new hydroxyapatite-alginate-gelatin biocomposite favor bone regeneration in a critical-sized calvarial defect model

**DOI:** 10.1590/0103-6440202405461

**Published:** 2024-05-10

**Authors:** Anderson Cunha dos Santos, Mauricio Andres Tinajero Aroni, Suzane Cristina Pigossi, Maria Eduarda Scordamaia Lopes, Paulo Sergio Cerri, Fúlvio Borges Miguel, Silvia Rachel de Albuquerque Santos, Joni Augusto Cirelli, Fabiana Paim Rosa

**Affiliations:** 1 Instituto de Ciências da Saúde(ICS), Universidade Federal da Bahia(UFBA), Salvador- BA, Brasil; 2 Centro Universitário Maria Milza(UNIMAM), Governador Mangabeira- BA, Brasil; 3 Departamento de Diagnóstico e Cirurgia - Periodontia, Faculdade de Odontologia de Araraquara, Universidade Estadual Paulista - UNESP, Araraquara, São Paulo, Brasil; 4 Universidad de Especialidades Espíritu Santo(UEES), Samborondón, Ecuador; 5 Departamento de Periodontia e Implantodontia, Faculdade de Odontologia, Universidade Federal de Uberlândia, Uberlândia, MG, Brasil; 6 Departamento de Morfologia e Clínica Infantil, Faculdade de Odontologia de Araraquara, Universidade Estadual Paulista - UNESP, Araraquara. São Paulo, Brasil; 7 LABIOMAT, Centro Brasileiro de Pesquisas Físicas(CBPF), Rio de Janeiro, RJ, Brasil.

**Keywords:** bone regeneration, alginates, hydroxyapatite

## Abstract

This study aimed to evaluate the osteogenic potential of hydroxyapatite (HA), Alginate (Alg), and Gelatine (Gel) composite in a critical-size defect model in rats. Twenty-four male rats were divided into three groups: a negative control with no treatment (Control group), a positive control treated with deproteinized bovine bone mineral (DBBM group), and the experimental group treated with the new HA-Alg-Gel composite (HA-Alg-Gel group). A critical size defect (8.5mm) was made in the rat's calvaria, and the bone formation was evaluated by in vivo microcomputed tomography analysis (µCT) after 1, 15, 45, and 90 days. After 90 days, the animals were euthanized and histological and histomorphometric analyses were performed. A higher proportion of mineralized tissue/biomaterial was observed in the DBBM group when compared to the HA-Alg-Gel and Control groups in the µCT analysis during all analysis periods. However, no differences were observed in the mineralized tissue/biomaterial proportion observed on day 1 (immediate postoperative) in comparison to later periods of analysis in all groups. In the histomorphometric analysis, the HA-Alg-Gel and Control groups showed higher bone formation than the DBBM group. Moreover, in histological analysis, five samples of the HA-Alg-Gal group exhibited formed bone spicules adjacent to the graft granules against only two of eight samples in the DBBM group. Both graft materials ensured the maintenance of defect bone thickness, while a tissue thickness reduction was observed in the control group. In conclusion, this study demonstrated the osteoconductive potential of HA-Alg-Gel bone graft by supporting new bone formation around its particles.

## Resumo

Este estudo teve como objetivo avaliar o potencial osteogênico de um compósito de hidroxiapatita (HA), alginato (Alg) e gelatina (Gel) em um modelo de defeito de tamanho crítico em ratos. Vinte e quatro ratos machos foram divididos em três grupos: um controle negativo sem tratamento (grupo controle), um controle positivo tratado com osso bovino desproteinizado (grupo DBBM) e o grupo experimental tratado com o novo compósito HA-Alg-Gel (grupo HA-Alg-Gel). Um defeito de tamanho crítico (8,5mm) foi feito na calvária dos ratos, e a formação óssea foi avaliada por análise de microtomografia computadorizada in vivo (µCT) após 1, 15, 45 e 90 dias. Após 90 dias, os animais foram eutanasiados e análises histológicas e histomorfométricas foram realizadas. Uma maior proporção de tecido mineralizado/biomaterial foi observada no grupo DBBM quando comparado aos grupos HA-Alg-Gel e controle na análise de µCT durante todos os períodos de análise. Entretanto, não foram observadas diferenças na proporção tecido mineralizado/biomaterial no dia 1 (pós-operatório imediato) em relação aos períodos posteriores de análise em todos os grupos. Na análise histomorfométrica, os grupos HA-Alg-Gel e controle apresentaram maior formação óssea do que o grupo DBBM. Além disso, na análise histológica, cinco amostras do grupo HA-Alg-Gal exibiram espículas ósseas formadas adjacentes aos grânulos do enxerto contra apenas duas das oito amostras do grupo DBBM. Ambos os materiais de enxerto garantiram a manutenção da espessura óssea do defeito, enquanto uma redução da espessura do tecido foi observada no grupo controle. Em conclusão, este estudo demonstrou o potencial osteocondutor do enxerto ósseo de HA-Alg-Gel, promovendo a formação de osso novo ao redor das suas partículas.

## Introduction

The treatment of extraction sockets with severe bone loss is considered a challenge in clinical practice. Severe periodontal disease, endodontic failure, or tooth fracture are common causes of extensive bone loss that prevent initial implant stabilization, as well as an adequate architecture of soft and hard tissues. In this context, alveolar ridge augmentation procedures are necessary to restore adequate bone dimensions to provide functional and aesthetic prosthetic rehabilitation ^1^.

Alveolar ridge augmentation involves grafting the sockets by using biomaterials, including autogenous, allogenous, xenogenic, and alloplastic bone substitutes, to ensure bone formation. Autogenous bone grafting is currently recognized as a gold standard for bone regeneration procedures due to its osteogenic and osteoinductive potential ^2^. However, the amount of bone graft is limited at the donor site, and the need to harvest bone from other sites increases the surgical morbidity associated with these procedures. In addition, the autogenous bone showed a tendency for rapid resorption that can result in the collapse of the space required for bony ingrowth ^2^. In this context, one xenogeneic graft comprised of deproteinized bovine bone mineral (DBBM) has been widely used as an alternative to autogenous bone grafting in treating ridge defects ^2^.

The main advantages of the bovine xenografts used as bone substitutes are unlimited availability and the absence of a second surgical procedure. Studies investigating the regeneration potential of the bovine xenografts showed that this material is osteoconductive, acting as a scaffold supporting new bone tissue formation in the extraction sockets ^3^ ^,^ ^4^. Moreover, once the DBBM particles are surrounded by new bone, they help tissue contour stabilization ^4^. However, the slow degradation of the biomaterial particles might also prevent their replacement by new bone. Besides, although xenogeneic bone grafts are biocompatible materials, the risk of disease transmission or hypersensitivity reaction cannot be completely discarded, in addition to the disadvantage of their higher costs ^5^.

As an alternative to xenogeneic biomaterials, several alloplastic materials in microspheres and granules format have been developed for bone regeneration due to their availability on a large scale and biocompatibility ^5^. Among the most used substrates in alloplastic material synthesis are bioceramics based on calcium phosphate, mainly hydroxyapatite (HA), due to its biocompatibility, bioactivity, and osteoconduction properties ^6^. Nevertheless, the poor mechanical properties are important limitations that require solutions. Moreover, the use of HA may be limited due to its slow absorption rate and low solubility ^7^. To overcome these limitations, developing composites of apatite polymers emerged as promising materials applied in biomedical engineering to form porous mineralized scaffolds. These materials showed both ceramic and polymer physicochemical properties in the same scaffold, which mimics the inorganic and organic phases of natural bone ^7^.

In this perspective, alginate (Alg), a natural anionic and hydrophilic polysaccharide composed of guluronic and mannuronic acids with excellent biocompatibility, has been widely used in composite synthesis. Alg is considered as non-toxic, non-immunogenic, and biodegradable ^8^. In addition to biocompatibility, the abundance of the source and the low prices make Alg-based composites promising materials for bone tissue regeneration ^8^. However, due to the lack of mechanical strength in the Alg scaffold to mimic the natural bone function, it has been combined with inorganic materials such as HA to enhance strength, as well as bone tissue formation ^9^. In vivo, studies showed that HA and Alg composites (HA-Alg) promoted bone formation in calvaria critical-sized bone defects with great potential for application as bone grafts in clinical areas ^7^ ^,^ ^10^ ^,^ ^11^ ^,^ ^12^.

Since native bone tissue is composed of organic collagen and inorganic HA crystals in an interconnected pore network structure, Gelatin (Gel), a natural water-soluble polymer derived from collagen by hydrolytic degradation, has also been utilized in composite materials synthesis. The gel is biocompatible, biodegradable, nonimmunogenic, and highly accessible ^13^ ^,^ ^14^. It can also form crosslinked hydrogel structures with excellent cell proliferation and differentiation properties ^15^ ^,^ ^16^. Thus, the HA granules encapsulation into the Alg and Gel hydrogel matrix might be crucial to enhance osseointegration, osteoconductivity, and structural stability to improve bone regeneration in the defect zone. Based on that, this study aimed to evaluate the osteogenic potential of an HA, Alg, and Gel (HA-Gel-Alg) composite in a critical-size defect model in rats.

## Materials and methods

### Study design

Twenty-four male rats (*Rattus norvegicus*) with 350 and 400g body weight were included in the study. The sample size calculation was performed using the statistical software G* Power (version 3.1.9.2). A minimum of 8 animals per group was defined for applying statistical tests, considering 80% power and 95% significance level. The number of animals required in this study was estimated based on the bone formation percentage (%) obtained by histomorphometry analysis in a previous study ^17^ considering an effect size f of 0.65 between the groups. The animals were fed a standard laboratory diet with ad libitum access to water in the animal facility of the School of Dentistry at Araraquara. All experiments were conducted in accordance with the guidelines established by the Brazilian Council of Animal Care (CONCEA) and approved by CEUA-UNESP 27/2016.

The rats (n=8) were randomly divided into three groups: Control group: negative control group with no treatment; DBBM group: positive control group, where bone defects were filled with deproteinized bovine bone mineral granules of 0.25mm - 1mm size (Bio-Oss®, Geisttlish Pharma, Wolhusen, Switzerland), and HA-Alg-Gel group; experimental group, where bone defects were filled with the new HA-Alg-Gel composite (HA 0.42 - 0.6 mm size granules encapsulated with Alg 1% and Gel 1%). The preparation and physicochemical characterization of the HA-Alg-Gel composites were reported in supplementary materials.

### Surgical procedures

General anesthesia was induced using intramuscular injections of ketamine hydrochloride (0.12 ml/kg body weight; Agener União, Brazil) and xylazine hydrochloride (0.06 ml/kg body weight; Bayer, Brazil). Surgeries were performed by using standard aseptic techniques. After shaving and preparing the frontoparietal region, it was made an incision over the interparietal suture. Bone defects were executed on each animal's central portion of the calvaria with an 8 mm-diameter trephine bur (Dentoflex®, São Paulo, Brazil) under copious saline irrigation. The resulting bone fragments were carefully elevated with a Freer elevator (Quinelato, Schobell Industrial, Brazil), thereby maintaining the integrity of the dura mater and brain. Bone defects in the Control group were filled with the blood clot only. Bone defects in the HA-Alg-Gel and DBBM groups received the respective bone graft granules.

The flaps were sutured with 4-0 vicryl® (polyglactin 910) and 4-0 mononylon (Ethicon, Johnson & Johnson, Brazil). In the immediate postoperative period, all animals received a subcutaneous injection of Tramadol (12.5 mg/kg; 8/8 hours for 2 days) diluted in saline solution (Tramal, União Química, Brazil).

### Micro-computed tomography analysis (µCT)

For the µCT analysis, the rats were sedated with ketamine hydrochloride (0.12 ml/kg body weight; Agener União, Brazil) and immobilized in the equipment saddle for image acquisition. The animals were scanned in the baseline and after 15, 45, and 90 postoperative days on a µCT in vivo analysis system (SkyScan 1176; 2000 x 2000 pixels, 1.0-mm Al filter, 65kV kilovoltage, 385µA milliampere and 320ms exposure time). The images were reconstructed using specific software (Nrecon 1.6.1.5; SkyScan). After reconstruction, the images were three-dimensionally repositioned using Dataviewer software and analyzed in the CTan software with the following parameters: circular ROI of 8 mm, 50 slides (18 µm of thickness each), and threshold of 62-255. The morphometric parameters obtained by CTan were: bone tissue volume (BV; mm^3^); bone volume/tissue volume (BV/TV; percentage (%)); bone contact surface (BS; mm^2^); bone surface/tissue volume (BS/TV; 1/mm).

### Histological and histomorphometric analysis

The animals were euthanized 90 days after surgery by anesthetic overdose. The specimens were reduced and immersed in 4% formaldehyde buffered with 0.1 M sodium phosphate at pH 7.2 for 72 hours. After fixation, the samples were decalcified with Ethylenediaminetetraacetic acid (EDTA 7%). The samples were splinted in the middle of the defect and then embedded in paraffin. Routine histological processing for light microscopy was carried out, and as 6-µm thick semi-serial starting from the middle region of the defect area, 18 semi-serial sections were obtained on each side toward the lateral aspect of the cranium (temporal bones) spanning 108 µm on each side of the defect. Six equally distant sections (18 µm) were stained with hematoxylin-eosin (HE) and 0.1% picrosirius-red solution (PR). The PR-stained sections were analyzed under polarized illumination using an Olympus microscope (Olympus BX-51, Tokyo, Japan).

The HE-stained sections were analyzed under a DIASTAR optical microscope (Leica Reichert & Jung products, Germany) coupled with a DXC-1107A/107AP digital camera (Sony Electronics, Japan). A researcher (P.S.C) blinded to the experimental groups analyzed the following parameters: mineralized bone quality, evidence of fibrotic tissue formation within the defect site, angiogenesis, inflammatory reaction, degradation, and material encapsulation.

Histomorphometric analysis was performed to assess the new bone tissue area formed in the bone defect edge in all samples. The margins of the original defect were identified, and the new bone formed in the defect edge was assessed. The margins of the original defect were identified visually based on the differences in collagen fiber arrangement between mature bone and newly formed bone. Moreover, five samples of the HA-Alg-Gal group and two samples in the DBBM group exhibited formed bone spicules adjacent to the graft granules. The bone area in contact with the granules was also measured in these samples. Five equally spaced (30μm distance between the cuts) histological sections were used from each sample for both analyses. The analysis was made by an image analysis software (Image J, Jandel Scientific, San Rafael, CA, USA) by an experienced examiner (A.C.S.) blinded to the groups.

Considering that newly formed bone during tissue repair is characterized by the irregular arrangement of collagen fibers in contrast to the lamellar arrangement typical of mature bone, PR-stained sections were analyzed under polarized illumination using an Olympus microscope (Olympus BX-51, Tokyo, Japan), focusing on the edges of the critical defects.

### Statistical analysis

The Kolmogorov-Smirnov test was used to assess the normality of the data. The two-way ANOVA followed by Tukey’s test was used to evaluate the µCT data. In addition, the one-way ANOVA followed by Tukey’s test was used to evaluate the histomorphometric data. All statistical analyses were performed using GraphPad Prism 5 (San Diego, CA), and the statistical differences were considered significant if their p values were <0.05 (p<0.05).

## Results

### Micro-computed tomography analysis (µCT)

**Figure 1:**
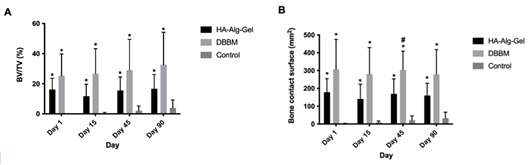
Microcomputed tomography analysis of HA-Alg-Gel, DBBM, and control group after 1, 15, 45, and 90 days of post-operative. A: Bone volume/Tissue volume [BV/TV; (%)] and B: Bone contact surface (BS; mm^2^); *p<0.05 about control group; #p<0.05 HA-Alg-Gel group about DBBM group.

**Figure 2:**
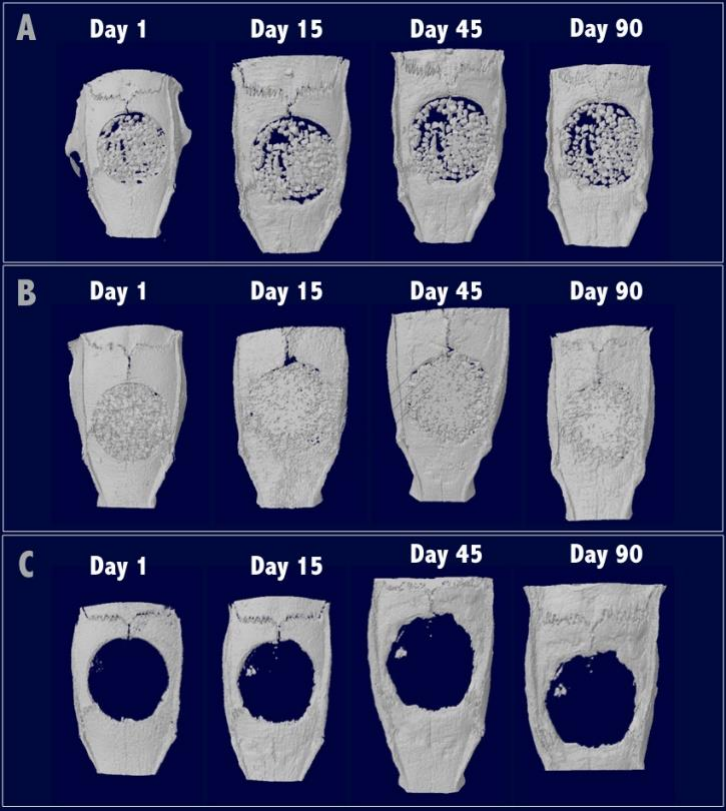
Microtomographic images of calvarial defects of all groups after 1, 45, and 90 days. A: HA-Alg-Gel group; B: DBBM group; C: Control group.

All animals were included in the analysis. The BV/TV analysis (%) demonstrated significantly higher mineralized tissue/biomaterial in the DBBM (25.32%±14.43) and HA-Alg-Gel (16.16%±7.46) groups compared to the Control group (0.0%±0.0) at day 1 (Figure 1A). No mineralized tissue was observed in the Control group at this time point. DBBM group showed higher BV/TV than the HA-Alg-Gel group but without statistical difference. The BV/TV on day 1 in both experimental groups corresponds to the graft granules, which suggests that a higher amount of DBBM graft granules remained within the bone defect after suturing compared to HA-Alg-Gel granules (Figure 2).

After 15 days, significantly higher mineralized tissue was observed in the DBBM group (26.68%±16.66) and HA-Alg-Gel (11.68%±7.98) groups compared to the Control groups (0.49%±0.45) (Figure 1A). Similarly, both DBBM and HA-Alg-Gel group showed significantly higher BV/TV after 45 (DBBM: 28.96%±20.53; HA-Alg-Gel:15.55%±8.91) and 90 days (DBBM: 32.67%±21.56; HA-Alg-Gel: 16.67%±9.44) compared to the Control group (2.05% ±3.17; 3.91%±5.37) (Figure 1A). The DBBM group also showed higher BV/TV than the HA-Alg-Gel group, but without a statistical difference, in both periods of analysis. No statistical difference was observed between the analyzed periods for any of the study groups, which suggests that most of the biomaterial granules were still inside the defects in both experimental groups after 90 days of postoperative.

The BS analysis (mm^2^) demonstrated significantly higher mineralized tissue surface in the DBBM (306.8mm^2^ ±168.2) and HA-Alg-Gel (165.3mm^2^ ±95.07) groups compared to the Control group (2.14 mm^2^±1.80) on day 1 (Figure 1B). The DBBM group showed higher BS than the HA-Alg-Gel group but without statistical difference. After 15 days, significantly higher BS in the DBBM group (280.3mm^2^±148.6) and HA-Alg-Gel (140.4mm^2^±82.56) was observed compared to the Control groups (9.18mm^2^±8.06). Both the DBBM (303.7mm^2^ ±105.4; 278.6mm^2^ ±138.9) and HA-Alg-Gel groups (169.3mm^2^±84.28; 159.8mm^2^±69.22) showed significantly higher BS compared to the Control group (21.51mm^2^ ±23.95; 32.08mm^2^ ±34.25) after 45 and 90 days. Moreover, the DBBM group showed significantly higher BS than the HA-Alg-Gel group after 45 days. No statistical difference was observed between the analyzed periods for any of the study groups (intragroup comparison).

### Histological analysis

**Figure 3:**
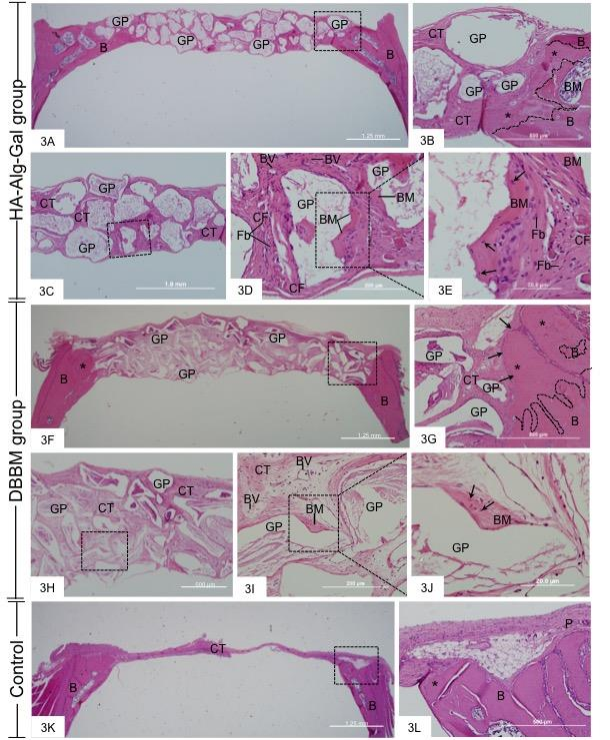
Light micrographs of sections stained with hematoxylin and eosin (HE) showing the critical defects made in calvarium from HA-Alg-Gel (Figs. 3A-3E), DBBM (Figs. 3F-3J) and Control (Figs. 3K and 3L) groups. In 4A, graft HA-Alg-Gel particles with varied size (GP) surrounded by connective tissue fill the critical bone defect. The irregular bone defect edges (B) are in contact with graft particles (GF). The 4B, outlined area of 3A, shows graft particles (GP) surrounded by connective tissue (CT) next to the bone defect edge (B). In the bone defect, a forming bone matrix (asterisks) is seen on the old bone (B). The dashed line delimits the old bone (pre-existent) from the newly formed bone. In 4C, a central portion of the critical defect shows several graft particles (GF) surrounded by fibrous connective tissue (CT). The outlined area is observed in 4D - bone matrix (BM) is observed adjacent to the graft particles (GP), which are surrounded by numerous fibroblasts (Fb) intermingled with bundles of collagen fibers (CF). BV, blood vessels. Fig. 4E, high magnification of the outlined area of 3D, shows a graft particle (GP) surrounded partially by bone matrix (BM). Some elliptical osteocytes (arrows) within lacunae are observed in the strongly eosinophilic (stained by eosin) and irregular spicule of bone matrix (BM). Figs. 4F-4J (DBBM group) - 4F shows a general view of a critical defect filled with graft particles (GP) with variable size and shape. Fig. 4G shows a high magnification of the outlined area in 3F. The dashed line indicates the new bone formed (asterisks) on the pre-existent bone (B) at the critical defect edge. DBBM particles (GP) surrounded by connective tissue (CT) are seen near the bone surface. A continuous layer of osteoblasts (arrows) covers the newly formed bone (asterisks). Figs. 4H-4J - show connective tissue (CT) and graft particles (GP) in the central portion of the bone defect. In Figs. 4I and 4J (high magnifications of the outlined area in 3H): an irregular layer of bone matrix (BM), containing entrapped cells (arrows), is adjacent to a graft particle (GP). The DBBM particles (GP) are surrounded by loose connective tissue (CT), containing some inflammatory cells, mainly macrophages, and blood vessels (BV). Figure 4K (control group): a thin layer of connective tissue (CT) fills the bone defect. In Fig. 3L, an outlined area of 3K, newly formed bone (asterisk) is apposed on the pre-existing bone (B) in the critical defect edge. P, periosteum.

All animals were included in the analysis. After 90 days, in the HA-Alg-Gal group, the defect was filled with irregularly shaped graft granules and connective tissue containing mainly fibroblasts and several blood vessels (Figures 3A-3E). These graft granules were usually surrounded by a well-defined layer of fibrous connective tissue with fibroblasts (Figures 3B-3E). In addition, some HA-Alg-Gal granules were partially covered by bone spicules with elliptical osteocytes inside the lacunae (Figures 3D and 3E). In the edge of the bone defect, newly formed bone with numerous and large osteocytes was seen. In this region, some bone spicules are projected towards graft granules (Figure 3B).

In the DBBM group, the defect was filled by the graft granules with irregular geometric shapes intermingled with connective tissue containing fibroblasts, macrophages, thin collagen fibers, and blood vessels (Figures 3F-3J). In the bone defect edges filled with DBBM granules, newly formed bone containing numerous osteocytes was observed. In addition, some graft granules were near the newly formed bone, which was covered by a continuous layer of osteoblasts (Figure 3G). Although a thin layer of collagen fibers was adjacent to the DBBM granules, inflammatory cells were often observed in the loose connective tissue among graft granules (Figure 3I). In addition, a dense collagen matrix containing elliptical (osteocyte-like) cells was seen adjacent to the DBBM granules (Figure 3I and 3J). In the control group, a continuous and thin layer of fibrous connective tissue was present, filling the entire bone defect (Figure 3K). Newly formed bone was restricted to the edges of the critical defects (Figure 3L).

**Figure 4:**
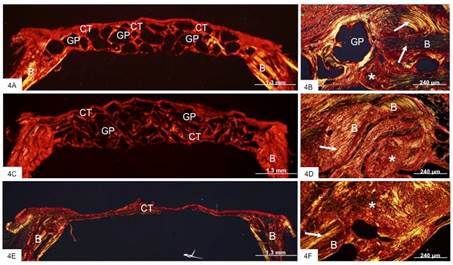
Light micrographs of sections stained with picrosirius-red and analyzed under polarized illumination showing the critical defects made in the rat calvarium from HA-Alg-Gel (Figs. 5A and 5B), DBBM (Figs. 5C and 5E) and Control (Figs. 5F and 5G) groups. Figs. 5A and 5C - show a general view of bone defects filled with graft particles (GP) and connective tissue (CT), which exhibits birefringent collagen (in red/orange color). B, bone of calvarium. Figs. 5B and 5D show the newly formed bone (asterisks) on the pre-existent bone (B) in the bone defects filled with graft particles. Note that pre-existent bone (B) exhibits collagen fiber lamellae (arrows), while in the newly formed bone (asterisks), the collagen fibers are randomly arranged. Figs. 5E and 5F (control group) - In 5E, a birefringent thin layer of fibrous connective tissue (CT) fills the entire bone defect. Fig. 5F: in the defect edge, newly formed bone (asterisks) on the mature, which contains collagen fiber lamellae (arrows), is seen.

The analysis of PR-stained sections under polarized illumination revealed the birefringent collagen fibers surrounding graft particles in HA-Alg-Gal and DBBM groups (Figures 4A and 4C). In contrast, in the control group, a thin layer of birefringent collagen was observed throughout the bone defect (Figure 4E). In addition, the newly formed bone at defect edges was recognized by the presence of birefringent collagen fibers randomly distributed on the mature bone, which showed collagen fiber lamellae with an organized arrangement (Figures 4B, 4D, and 4F). A newly formed bone matrix exhibited collagen fibers randomly arranged.

### Histomorphometric analysis

**Figure 5:**
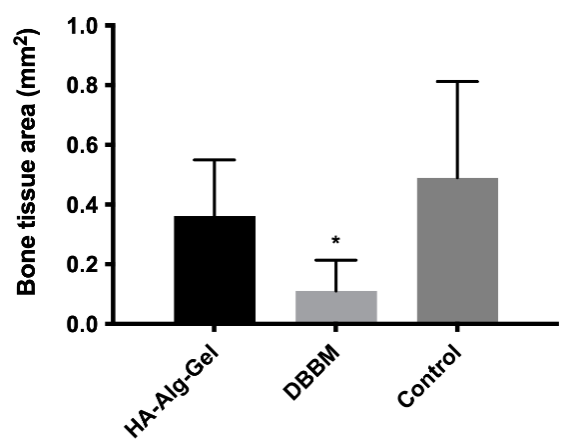
New bone area of HA-Alg-Gel, DBBM, and control group after 90 days of post-operative (histomorphometric analysis); *p<0.05 about control group.

In the histomorphometric analysis, higher bone formation was observed in the HA-Alg-Gal group (0.3625 mm^2^±0.1870) compared to the DBBM group (0.1100 mm^2^±0.1036), but without a statistical difference (Figure 5). Significantly higher bone formation was observed in the Control group (0.4900 mm^2^±0.3220) compared to the DBBM group after 90 days (Figure 5). No significant difference was found between the HA-Alg-Gal and Control groups (Figure 5).

Five samples of the HA-Alg-Gal group exhibited formed bone spicules adjacent to the graft granules against only two of eight samples in the DBBM group. Moreover, the bone area in contact with the granules in the HA-Alg-Gal group (4.829.711 µm^2^) was about 7.5 folds higher than those from the DBBM group (625.311 µm^2^).

## Discussion

In the present study, a novel alloplastic bone substitute composed of HA, Alg, and Gel (HA-Alg-Gel) was proposed as an alternative to xenograft materials, and its osteoconductive and biodegradation property was evaluated by measuring mineralized tissue in critical-size defects in rat calvaria using µCT and histological/histomorphometric analyses. The HA-Alg-Gel graft bone was compared to a commercial bone substitute (Bio-Oss®) composed of bovine hydroxyapatite with osteoconductive properties that support new bone formation in dentistry bone regeneration procedures. Bio-Oss® is currently the biomaterial with the most clinical and scientific evidence in the literature for buccal applications ^18^ ^,^ ^19^. Moreover, the calvarial defect was used to simulate the sinus cavity or a large alveolar defect since the calvarial bone especially consists of cortical bone with minimal bone marrow cells ^20^.

The bone repair mechanism is a dynamic, temporal, and complex phenomenon consolidated by bone regeneration under physiological conditions ^7^. However, the bone regenerative potential is compromised in bone defects with critical dimensions and the tissue repair usually occurs by fibrosis ^21^ ^,^ ^22^. In the present study, an 8.5 mm critical size defect was made in the rats' calvaria, resulting in a minimum bone formation in the Control group in all periods of µCT analysis and after 90 days in the histomorphometric analysis. Furthermore, the histological analysis showed that bone formation was limited to the edges of the defect, and the defect was all filled by fibrous tissue in the Control group, as observed in other studies ^7^ ^,^ ^21^ ^,^ ^22^.

In the grafted groups, the biomaterial filled the defects and was maintained for all the study extensions, as confirmed by µCT and histological analyses. In general, higher mineralized tissue was observed in the DBBM group compared to the HA-Alg-Gel and Control groups in the µCT analysis in all periods of analysis. In addition, no differences were observed in the amount of mineralized tissue/biomaterial observed on day 1 (immediate postoperative) in comparison to late periods of analysis for all groups. These results suggest that the mineralized tissue observed in µCT analysis, when analyzed together with histological analysis, mostly corresponds to the graft granules filled the bone defect. It was clear that until the last period assessed (90 days), both biomaterials kept filling the bone defect in all its extensions, as observed in another study ^23^. However, the histomorphometric analysis showed less bone formation in the defect edge for the DBBM group compared to the HA-Alg-Gel and Control groups. Similarly, as was found in the present study, other studies showed reduced new bone formation in critical-size defects filled with DBBM grafts against initial expectations ^23^ ^,^ ^24^.

The HA-Alg-Gel granules graft showed similar bone formation in the defect edge compared to the Control group in the histomorphometric analysis. However, the histological analysis showed that HA-Alg-Gel graft granules ensured the maintenance of defect bone thickness while a tissue thickness reduction was observed in the Control group. Moreover, the histological analysis revealed that bone formation was observed mainly on the edges of the defect. However, bone spicules adjacent to the graft granules were observed in five samples of the HA-Alg-Gel group (against only two of eight samples in the DBBM group). This suggests that the biomaterial was osteoconductive. Similar results were observed in other studies that evaluated hydroxyapatite and alginate composites in the critical size defect model ^7^ ^,^ ^10^ ^,^ ^11^. The bone formation adjacent to the graft granules could be associated with the alginate phase of the composite that forms highly anionic polymers (rich in carboxylates), with the potential to absorb important molecules for osteoblast differentiation ^11^. Besides, the Alg in the granules graft is dissolved and reabsorbed in contact with fluids and tissues by local enzymes. This dissolution allows a gradual release of inorganic components of the composite, especially ions of Ca and PO4, present in the HA crystals ^7^. Finally, it can also be attributed to the HA and alginate/gelatin characteristics that mimic natural bone tissue's inorganic and organic phases.

The histological analysis showed connective tissue around the granules of both groups. Similar results were observed by Kato, Lemler ^20^ in a study evaluating the Bio-Oss Collagen in the calvarial bone defect. According to these authors, the newly formed dense connective tissue associated with residual particles of the Bio-Oss material revealed that the particles were merely surrounded but not absorbed by the newly formed tissue. It is important to reinforce that, although the slowly resorbing bone substitutes ensure the maintenance of the augmented tissue volume over the long term, a slow degradation might prevent new bone formation ^25^.

Histological analysis showed the presence of newly formed blood vessels surrounding the DBBM and HA-Alg-Gel granules, which reveals that both materials were biocompatible. Furthermore, inflammatory cells associated with the DBBM granules were compatible with those expected when a biomaterial is implanted in the organism ^26^. This finding reinforces the biocompatibility of both biomaterials since no rejection by acute exacerbated inflammation was observed after 90 days of postoperative ^26^.

In conclusion, the osteoconductive property of HA-Alg-Gel bone graft seems to be superior to DBBM granules by supporting higher new bone formation around its particles. On the other hand, higher mineralized tissue/biomaterial was observed in the DBBM graft. In addition, both graft materials ensured the maintenance of defect bone thickness, while a tissue thickness reduction was observed in the control group. However, additional studies must be performed to better explore and confirm these results.

## References

[1] Barone A, Borgia V, Covani U, Ricci M, Piattelli A, Iezzi G (2015). Flap versus flapless procedure for ridge preservation in alveolar extraction sockets: a histological evaluation in a randomized clinical trial. Clin Oral Implants Res.

[2] Song YW, Jung HJ, An YZ, Jung UW, Lee JS (2021). Addition of autogenous bone chips to deproteinized bovine bone mineral does not have additional benefit in lateral ridge augmentation-A preclinical in vivo experimental study. Clin Oral Implants Res.

[3] Schulz MC, Kallweit MB, Kallweit S, Koch R, Lauer G, Mai R (2016). Autogenous bone and a bovine bone substitute for ridge preservation: preliminary clinical and histologic findings. Aust Dent J.

[4] Araujo MG, Lindhe J (2009). Ridge preservation with the use of Bio-Oss collagen: A 6-month study in the dog. Clin Oral Implants Res.

[5] Girish Kumar N, Chaudhary R, Kumar I, Arora SS, Kumar N, Singh H (2018). To assess the efficacy of socket plug technique using platelet rich fibrin with or without the use of bone substitute in alveolar ridge preservation: a prospective randomised controlled study. Oral Maxillofac Surg.

[6] Cecoltan S, Stancu IC, Dragusin DM, Serafim A, Lungu A, Tucureanu C (2017). Nanocomposite particles with improved microstructure for 3D culture systems and bone regeneration. J Mater Sci Mater Med.

[7] Santos GGD, Vasconcelos LQ, Poy S, Almeida RDS, Barbosa AA, Santos SRA (2019). Influence of the geometry of nanostructured hydroxyapatite and alginate composites in the initial phase of bone repair1. Acta Cir Bras.

[8] Venkatesan J, Bhatnagar I, Manivasagan P, Kang KH, Kim SK (2015). Alginate composites for bone tissue engineering: a review. Int J Biol Macromol.

[9] Olderoy MO, Xie M, Andreassen JP, Strand BL, Zhang Z, Sikorski P (2012). Viscoelastic properties of mineralized alginate hydrogel beads. J Mater Sci Mater Med.

[10] Rossi AL, Barreto IC, Maciel WQ, Rosa FP, Rocha-Leao MH, Werckmann J (2012). Ultrastructure of regenerated bone mineral surrounding hydroxyapatite-alginate composite and sintered hydroxyapatite. Bone.

[11] De Paula F, Barreto I, Rocha-Leão M, Borojevic R, Rossi A, Rosa F (2009). Hydroxyapatite-alginate biocomposite promotes bone mineralization in different length scales in vivo. Frontiers of Materials Science in China.

[12] dos Anjos Ribeiro IÍ, dos Santos Almeida R, da Rocha DN, da Silva MHP, Miguel FB, Rosa FP (2014). Biocerâmicas e polímero para a regeneração de defeitos ósseos críticos. Revista de Ciências Médicas e Biológicas.

[13] Kuijpers A, Engbers G, Feijen J, De Smedt S, Meyvis T, Demeester J (1999). Characterization of the network structure of carbodiimide cross-linked gelatin gels. Macromolecules.

[14] Bae SK, Sung T-H, Kim J-D (2002). A soft-tissue gelatin bioadhesive reinforced with a proteinoid. Journal of adhesion science and technology.

[15] Sarker A, Linh NTB, Jung HI, Seo HS, Lee BT (2014). Fabrication of recombinant human bone morphogenetic protein-2 coated porous biphasic calcium phosphate-sodium carboxymethylcellulose-gelatin scaffold and its In vitro evaluation. Macromolecular Research.

[16] Van Vlierberghe S, Dubruel P, Schacht E (2011). Biopolymer-based hydrogels as scaffolds for tissue engineering applications: a review. Biomacromolecules.

[17] Quinlan E, Lopez-Noriega A, Thompson E, Kelly HM, Cryan SA, O'Brien FJ (2015). Development of collagen-hydroxyapatite scaffolds incorporating PLGA and alginate microparticles for the controlled delivery of rhBMP-2 for bone tissue engineering. J Control Release.

[18] Aludden HC, Mordenfeld A, Hallman M, Dahlin C, Jensen T (2017). Lateral ridge augmentation with Bio-Oss alone or Bio-Oss mixed with particulate autogenous bone graft: a systematic review. Int J Oral Maxillofac Surg.

[19] Jensen T, Schou S, Stavropoulos A, Terheyden H, Holmstrup P (2012). Maxillary sinus floor augmentation with Bio-Oss or Bio-Oss mixed with autogenous bone as graft: a systematic review. Clin Oral Implants Res.

[20] Kato E, Lemler J, Sakurai K, Yamada M (2014). Biodegradation property of beta-tricalcium phosphate-collagen composite in accordance with bone formation: a comparative study with Bio-Oss Collagen(R) in a rat critical-size defect model. Clin Implant Dent Relat Res.

[21] Miguel FB, Barbosa AA,, Marcantonio E,, Goissis G, Rosa FP (2006). Morphological assessment of the behavior of three-dimensional anionic collagen matrices in bone regeneration in rats. J Biomed Mater Res B Appl Biomater.

[22] Cardoso AK, Barbosa Ade A, Miguel FB, Marcantonio E E, Farina M, Soares GD (2006). Histomorphometric analysis of tissue responses to bioactive glass implants in critical defects in rat calvaria. Cells Tissues Organs.

[23] Grossi-Oliveira G, Faverani LP, Mendes BC, Braga Polo TO, Batista Mendes GC, de Lima VN (2020). Comparative Evaluation of Bone Repair with Four Different Bone Substitutes in Critical Size Defects. Int J Biomater.

[24] Tovar N, Jimbo R, Gangolli R, Perez L, Manne L, Yoo D (2014). Evaluation of bone response to various anorganic bovine bone xenografts: an experimental calvaria defect study. Int J Oral Maxillofac Surg.

[25] Park CH, Rios HF, Jin Q, Sugai JV, Padial-Molina M, Taut AD (2012). Tissue engineering bone-ligament complexes using fiber-guiding scaffolds. Biomaterials.

[26] Anderson JM, Rodriguez A, Chang DT (2008). Foreign body reaction to biomaterials. Semin Immunol.

